# Hydrogen: a review of the effects and underlying mechanisms in oral diseases

**DOI:** 10.3389/fphar.2026.1902221

**Published:** 2026-09-03

**Authors:** Zhaogang Liu, Yuexiu Li, Ying Liu, Zhentao Zhao, Li Zhang, Chenxin Ge, Jing Gao

**Affiliations:** 1 Department of Stomatology, The Affiliated Taian City Central Hospital of Qingdao University, Taian, China; 2 School and Hospital of Stomatology, Liaoning Provincial Key Laboratory of Oral Diseases, China Medical University, Shenyang, China; 3 School of Stomatology, Shandong Medical and Pharmaceutical University, Yantai, China

**Keywords:** antibacterial, anti-inflammatory, hydrogen, mucosal healing, oral diseases

## Abstract

Oral diseases can affect the quality of life of patients and cause secondary systemic diseases, thereby harming systemic health. Hydrogen has been shown to exhibit anti-oxidant stress, anti-inflammatory and anti-apoptosis and other effects, indicating its therapeutic significance for many kinds of diseases. Hydrogen administered through different methods can effectively reduce plaque biofilm formation, resist oxidative stress, inhibit the inflammatory response, and promote tissue repair and regeneration. This review summarizes the primary strategies for and research on hydrogen therapy, as well as the application of hydrogen in oral diseases such as dental caries, periodontitis, peri-implantitis, oral mucosal diseases, jaw diseases, oral and maxillofacial tumors, and trigeminal neuralgia. We highlight the regulatory effects of hydrogen on the functional states and interactions of common oral disease pathogens and various cells, including immune cells, osteoblast-associated cells, osteoclasts, and fibroblasts, and summarize the involved signaling pathways. In addition, this paper discusses the problems and shortcomings of the existing research, and highlights the directions for future development to provide the basis and support for future investigations into the mechanisms through which hydrogen exerts antioxidant stress and anti-inflammatory activity, promotes mucosal healing, enhances bone regeneration, as well as alleviates neuropathic pain.

## Graphical Abstract

**Figure:**
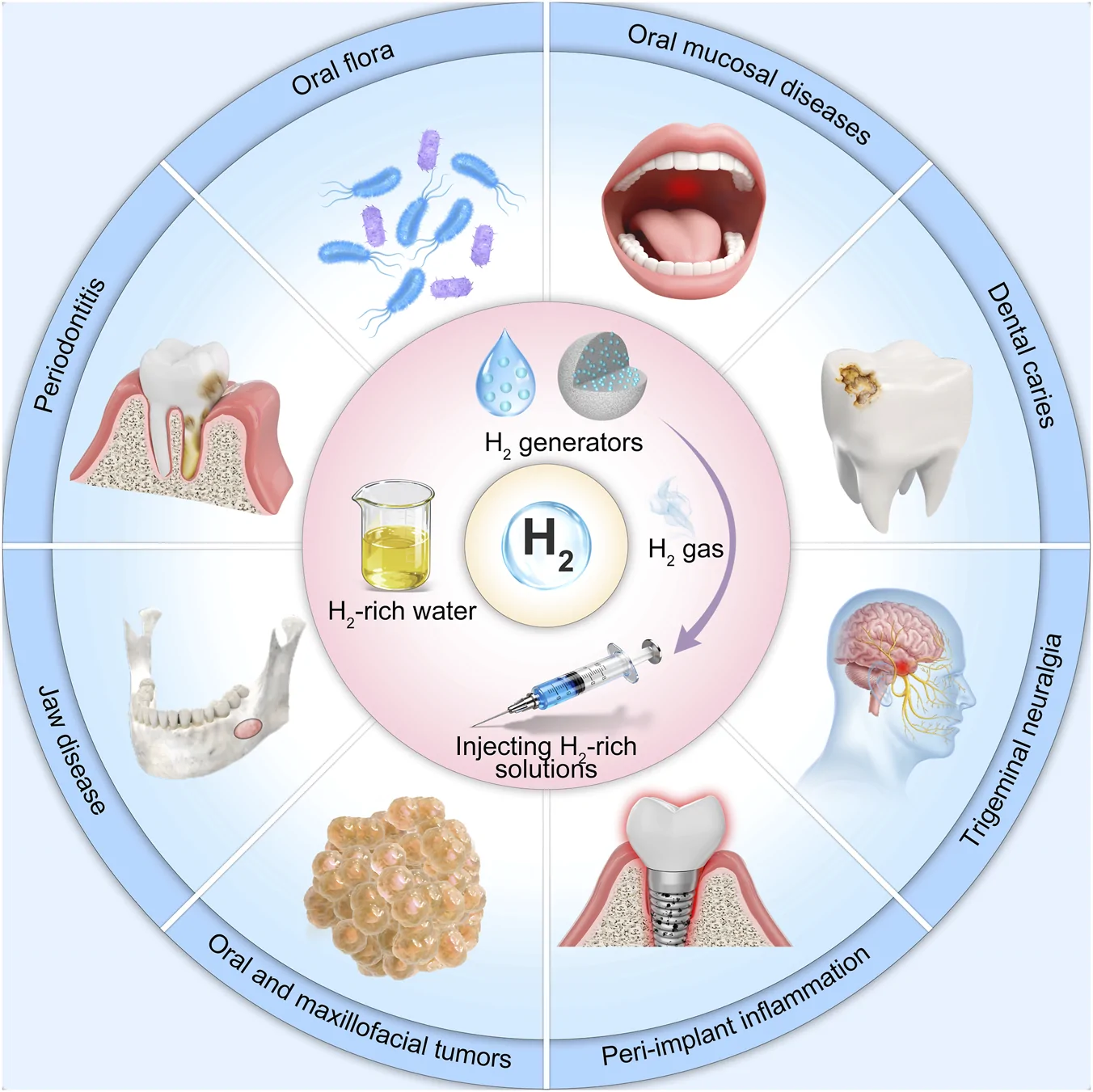

## 1 Introduction

Hydrogen (H_2_) is a colorless, odorless gas with a small molecular weight that can rapidly penetrate biological membranes to reach the nucleus and mitochondria to exert its effects. Hydrogen is a promising new medical gas with unique antioxidant stress, anti-inflammatory, antibacterial, and anti-apoptotic activities, as well as the ability to regulate of autophagy and preserve mitochondrial function and the blood-brain barrier (Chen et al., 2025; Chawla, 2025). Hydrogen has been proven to prevent brain damage attributable to various causes (Sato et al., 2025), alleviate significantly septic myocardial dysfunction (Cui et al., 2024), vascular dysfunction (Mu et al., 2022), as well as ischemia-reperfusion-induced ovary injury (Jiang et al., 2026), and protect against the progression of neurodegenerative diseases (Abdul-Nasir et al., 2025). Notably, hydrogen can selectively counteract deleterious reactive oxygen species (ROS) without affecting other physiologically functional ROS in the body (Meng et al., 2025; Kura and Slezak, 2024; Wang B. et al., 2022). Zhai et al. (2013) showed that lactulose induces the production of endogenous hydrogen, which increases superoxide dismutase activity while reducing the levels of malondialdehyde (MDA), 8-hydroxydeoxyguanosine (8-OHdG) and oxidative stress markers in the nucleus tractus solitarius, thereby alleviating oxidative stress. Zheng and Yu (2012) confirmed that hydrogen can inhibit the infiltration of neutrophils and macrophages in lung tissue.

With rapid advancements in hydrogen medicine, research on hydrogen has expanded exponentially across many fields. However, most studies are still limited to describing effect phenomena, and the molecular mechanisms underlying the biological effects of hydrogen remain unclear. In addition, research on hydrogen in oral diseases is particularly limited.

The oral cavity is an important structure connecting the human body and the external environment (Chen et al., 2023). Many microbes in the external environment can easily enter the oral cavity and reproduce there, thereby forming a unique oral microecosystem (Azuma et al., 2014). The plaque biofilm is the initiating factor for various oral diseases (Hu et al., 2025). If good oral hygiene is not maintained, bacteria can attach to the tooth surface and form dental plaque biofilm. This plaque secretes a variety of virulence factors that act on tooth surfaces and within the gingival sulcus or periodontal pocket and cause oral diseases (Toker et al., 2017; Li et al., 2022). Moreover, oxidative stress is an important factor leading to inflammatory diseases. The continuous accumulation of ROS further promotes the release of relevant pro-inflammatory factors, such as interleukin (IL)-1β and IL-6, causing progression of oral diseases such as periodontitis, periapical periodontitis, and peri-implantitis (Wang et al., 2024; Guo et al., 2024). These oral diseases can lead to tissue damage, bone-structure destruction, and tooth loss, seriously affecting the quality of life of patients. Previous studies have shown that hydrogen inhibits oxidative stress and related inflammatory responses caused by these oral diseases, producing an inflammatory microenvironment conducive to tissue repair, thereby promoting bone healing and wound repair (Tan et al., 2026; Xu et al., 2026).

In summary, dental plaque, oxidative stress and secondary inflammatory response are closely related to the occurrence and development of oral diseases (Ghensi et al., 2025; Kim et al., 2023). The excellent anti-inflammatory effect, anti-oxidative stress properties, and antibacterial properties of hydrogen indicate that hydrogen is a promising medical gas for oral diseases. Table 1 summarizes the latest findings regarding the effects of hydrogen on oral diseases.

**TABLE 1:** The latest reports regarding the effects of hydrogen on oral diseases.

The administration of hydrogen route	Study object	Cell line	Index/signaling pathway	Outcome	References
Drinking HW	Thirteen volunteers (3 women, 10 men, 22–40 years old) with periodontitis	NA	Serum ROM (−)	Drinking HW can enhance the effects of non-surgical periodontal treatment	(Azuma et al., 2015)
HW -pretreatment	NA	HGF cells	Serum ROM (−) GSH (+)	HW can increase intracellular antioxidative capacity	(Xiao and Miwa, 2016)
Drinking HW	Obese rats	NA	8-OHdG (−)	HW can suppress gingival oxidative stress and alveolar bone resorption	(Yoneda et al., 2017)
Drinking HW	Four-month-old male rats	NA	8-OHdG (−) NLRP3, caspase-1, ASC and IL-1b (+) NF-κB (−)	HW has anti-aging effects on periodontal oxidative damage	(Tomofuji et al., 2014)
Drinking HW	A rat model of periodontitis	NA	ROM, 8-OHdG and nitrotyrosine (−); GSH/GSSG (+) The MAPK pathway (−)	HW can suppress periodontitis progression by decreasing gingival oxidative stress	(Kasuyama et al., 2011)
HW	Twenty chronic periodontitis patients	NA	NA	HW has an antibacterial effect on microorganisms associated with chronic periodontitis	(Nayak et al., 2021)
H_2_ gas	NA	HGE cells	IL-1α and IL-6 (−)	H_2_ can alleviate periodontal diseases	(Saitoh et al., 2022)
HW	NA	HGF	NA	HW increased the migratory capacity, and showed an antioxidative potential on HGF	(Bhatt et al., 2023)
Drinking HW	Twenty-four male Wistar rats	NA	Nrf2, HO-1, NQO-1 (+); iNOS, nitrotyrosine (−); IL-6, TNF-α, IL-1β, MIP-1*α* (−); TGF-*β*1, FGF7, VEGF, *α*-SMA (+) The Nrf2/antioxidant defense pathway (+)	HW benefits the wound healing process	(Tamaki et al., 2016)
Hydrogen-rich medium/saline	A rat model of ORNJ	BMSCs	*α*-SMA (+)	Hydrogen may represent a strategy for the prevention and treatment of ORNJ.	(Chen et al., 2020)
Drinking HW	A rat model of periodontitis	NA	TNF-α, IL-1β, IL-6 (−); IL-10 (+)	HRW may be used to prevent or ameliorate periodontitis	(Bai et al., 2024)
HW	Beagle dog model of peri-implantitis	NA	IL-1, IL-6, and MMP-8 (−)	The use of HW for treating peri-implantitis showed promise and effectiveness	(Yiwei Zhao et al., 2023)
A hydrogen-producing Si-based agent	A rat model of TN	NA	The NLRP3-caspase-1-GSDMD pathway	Hydrogen can reduce the incidence of nerve demyelination	(Mu et al., 2023)

HW, hydrogen-rich water; HGF, human gingiva fibroblasts; ROM, reactive oxygen metabolites; GSH, glutathione; GSSG, Oxidized Glutathione; ORNJ, osteoradionecrosis of the jaw; TN, Trigeminal neuralgia; BMSCs, Bone marrow-derived mesenchymal stem cells; HGE, human gingival epithelium progenitor; 8-OHdG, 8-hydroxydeoxyguanosine; NLRP, Nod-like receptor protein; ASC, apoptosis-associated speck-like; IL, interleukin; ROO-, the peroxyl radical; ORAC-capacity, scavenging activity.

Despite the notable advances in the effectiveness and underlying mechanisms of hydrogen therapy, the current understanding of the exact mechanisms and optimal mode of administration of hydrogen treatment for oral diseases remain limited. This review summarizes the existing routes of hydrogen administration and the effects of hydrogen on the oral flora. Furthermore, we review the applications and mechanisms of hydrogen in various oral diseases and discuss the existing problems and challenges as well as the paths for future research and applications.

## 2 Hydrogen therapy strategies

Hydrogen therapy has recently emerged as a promising therapeutic strategy, garnering increasing attention across various medical disciplines (Maruyama et al., 2025; Wu et al., 2025). With advancements in gas extraction technology and the development of multidisciplinary comprehensive treatment, the administration methods and therapeutic effects of hydrogen have been widely studied by researchers. At present, the main methods of administering hydrogen gas for treating oral diseases include inhaling hydrogen gas, drinking or gargling hydrogen-rich water (HW), injecting hydrogen-rich solutions, and using drugs or medical materials as hydrogen generators for targeted-delivery and sustained release of hydrogen.

### 2.1 Inhaling hydrogen gas

Hydrogen inhalation can be used to treat brain injury (Nakagawa et al., 2026), vascular diseases, and cancer (Li D. et al., 2025). However, hydrogen gas can spontaneously ignite within the concentration range of 4%–74.2%, and it shows the risk of mixing with other gases during inhalation. In addition, hydrogen shows high diffusion, low aqueous solubility, and dose-dependent effects, which limit the efficacy of inhalation therapy (Zhu et al., 2022). These defects also pose safety and operational issues for research, limiting the clinical application of inhaling hydrogen gas.

### 2.2 Drinking or gargling HW

HW is a supersaturated solution formed by dissolving molecular hydrogen gas in water under high pressure. Hydrogen molecules are very small, so they can easily penetrate water and remain dissolved for a period of time (Tan et al., 2026). Under routine experimental conditions, drinking HW or hydrogen-containing solutions exhibits lower operational risks compared with inhaling hydrogen gas. For this reason, hydrogen-rich solutions have been increasingly explored as an adjuvant intervention for multiple disorders (Ji et al., 2024; Wu et al., 2022). In the past few decades, the effects of drinking HW have been studied in animals and humans, and it has shown antioxidant, anti-inflammatory, and anti-apoptotic effects (He et al., 2025; Li G. et al., 2025).

### 2.3 Injecting hydrogen-rich solutions

Although hydrogen administration by inhalation and drinking is convenient, the intake dose to a specific target area with these approaches is limited. Hydrogen-soluble saline can deliver dissolved molecular hydrogen *via* injection routes (Li G. et al., 2025; Shibuya et al., 2025). However, due to the invasive nature of hydrogen-dissolved saline injections, it is an inconvenient approach for daily hydrogen therapy. In addition, frequent injection of hydrogen-dissolved saline poses a risk of cross-infection, and high hydrogen concentration in blood vessels poses potential hazards (Shibuya et al., 2025; Tian et al., 2021).

### 2.4 Other strategies for hydrogen therapy

The non-targeted accumulation and high diffusivity characteristics of hydrogen limit the ability to achieve targeted, accumulation at high concentrations at lesion sites through conventional oral and inhalation methods, resulting in limited therapeutic effects (Kong et al., 2019; Liu et al., 2022). Therefore, a local hydrogen generator that can continuously provide hydrogen is urgently needed to achieve high bioavailability and efficiency in the treatment of certain specific diseases.

Responsive medicines and materials provide an important platform for achieving targeted and efficient hydrogen delivery to disease tissues, thereby improving the therapeutic effects of hydrogen. He et al. (Zhao et al., 2018) utilized palladium nanoparticles as carriers and responsively release hydrogen under near-infrared light irradiation, achieving combined hydrogen and thermal therapy for tumors guided by photoacoustic imaging. Photocatalytic hydrolysis for hydrogen production has also been used to achieve sustained and controllable hydrogen release. Wan et al. (2017) synthesized the nanoreactor that simulated photosynthesis to produce high levels of hydrogen and increase its local concentration to therapeutic levels.

Magnesium (Mg) has attracted attention as a potential hydrogen generator, because of its strong reduction properties, which can react with H_2_O and spontaneously produce hydrogen gas (Xu et al., 2021; Wan et al., 2018). Various magnesium-containing hydrogen generators have been developed, such as magnesium discs, magnesium hydride (MgH_2_), magnesium-hyaluronic acid micromotor (Mg-HA motor), magnesium-containing high polymers, and microneedle patches loaded with magnesium (Kong et al., 2019; Xu et al., 2021; Wan et al., 2018; Mei et al., 2024). In addition, nano-silicon (Si) has been shown to safely and continuously generate hydrogen in the alkaline environment of the intestine. Therefore, silicon-based drugs that can generate large amounts of hydrogen have been developed to treat various diseases (Wei K. et al., 2023; Johnsen et al., 2022).

## 3 Effects of hydrogen on oral flora

The dental plaque is the factor initiating a variety of oral diseases. The formed dental plaque on the tooth surfaces and within the gingival sulcus or periodontal pocket continuously secrete virulence factors, which can stimulate the surrounding tissues and cells to produce various inflammatory mediators. Therefore, inhibiting dental plaque formation is crucial for the treatment of oral diseases and the maintenance of oral health.

Caries, periodontal disease and peri-implantitis are prevalent bacterial oral diseases. *Streptococcus mutans* and *Streptococcus sobrinus* are the major pathogens in human dental caries and are considered to be the main cariogenic bacteria in oral streptococci (Sarah Samson et al., 2025). Studies summarizing pathogenic bacteria in periodontitis and peri-implantitis, have shown that these conditions share many of the same pathogenic bacteria. Among these, the most common were *Porphyromonas*, *Actinobacteria*, Streptococcaceae*,* and *Treponema* (Sedghi et al., 2021; Cui et al., 2025). Hydrogen can effectively inhibit the formation of oral plaque biofilms, prevent and control inflammatory damage, and help maintain oral health. HW has demonstrated antibacterial activity against oral bacteria such as *S. mutans, Fusobacterium nucleatum, Porphyromonas gingivalis,* and *Tannerella forsythia* (Pathak et al., 2021). Moreover, other studies have shown that HW effectively inhibites the proliferation of *S. mutans*, *P. gingivalis*, and *Aggregatibacter actinomycetemcomitans* (Hu et al., 2025; Bai et al., 2024; Makhija et al., 2023).

**FIGURE 1:**
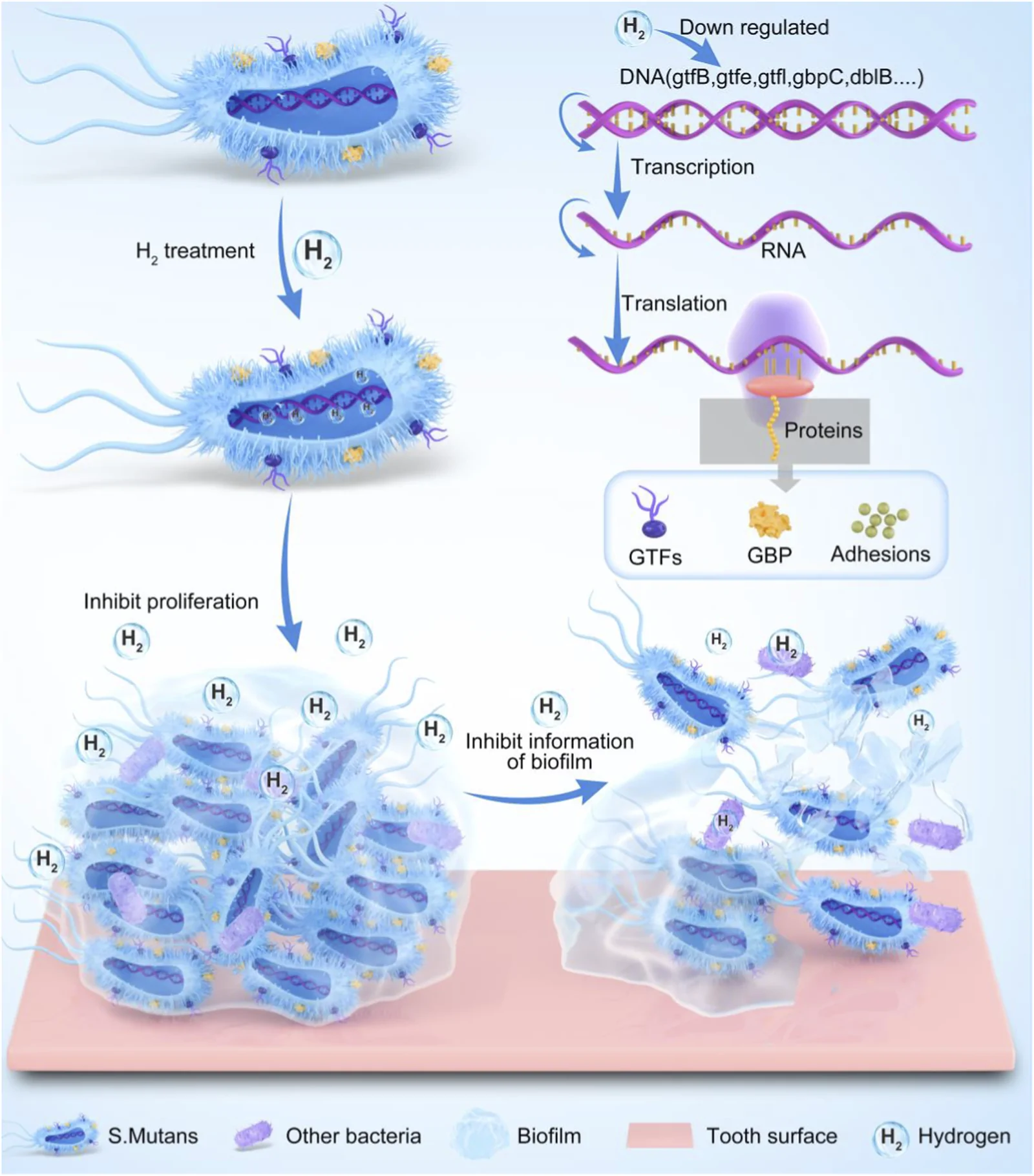
Schematic illustration of the antibacterial mechanism of hydrogen. Hydrogen exerts antibacterial effects on oral bacteria through two primary approaches: (1) Hydrogen can inhibit the mRNA expression levels of glycosyltransferases (gtfB, gtfc, and gtfl) and GBPs (gbpC, dblB). (2) Hydrogen can inhibit the proliferation of bacteria.

*Streptococcus mutans* can form glycosyltransferases (GTFs) to synthesize extracellular polysaccharides, which help them firmly adhere to teeth and promote tight cell aggregation. The binding of β-glucan-binding protein (GBP) to insoluble β-glucan is a crucial step in bacterial biofilm formation (Wang R. et al., 2022). Hydrogen can inhibit the mRNA expression of glycosyltransferases (gtfB, gtfc, and gtfl) and GBPs (gbpC, dblB), thereby inhibiting the formation of biofilms (Kim et al., 2017; Liu et al., 2023). In addition, HW can achieve antibacterial effects by inhibiting the proliferation of bacteria. A schematic illustration of the antibacterial mechanisms of hydrogen is shown in Figure 1. The colony-forming units of *Streptococcus salivarius* were significantly reduced after using HW for oral flushing (Nayak et al., 2021; Li G. et al., 2025). Exposing cariogenic bacteria (*S. mutans* and *S. sobrinus*) to HW for short-term can effectively inhibit bacterial growth and reduce the number of colony forming units (Liu et al., 2023). Table 2 summarizes the latest findings regarding the effects of hydrogen on oral flora involved in oral diseases.

**TABLE 2:** The latest reports regarding the effects of hydrogen on oral flora involved in oral diseases.

The administration of hydrogen route	Oral flora	Antimicrobial mechanism	Effects	Reference
HW	Streptococcal biofilm	Glucosyltransferases (gtfB, gtfc, and gtfI) and glucanbinding proteins (gbpC, dblB) (−)	Hydrogen can suppress biofilm development	(Kim et al., 2017)
HW	*S. mutans, F. nucleatum* and *P.gingivalis* and *tannerella forsythia*	NA	Hydrogen reduced the growth of all periodontopathogens, and that of aerobic and anaerobic bacteria in saliva	(Lee and Choi, 2006)
HW	*S. mutans*, F. *nucleatum* and *P.gingivalis*	NA	HW may have a positive impact on oral hygiene by helping to remove cariogenic bacteria and periodontopathogens	(Lee and Baek, 2013)
HW	Aerobic and anaerobic organisms associated with chronic periodontitis	NA	HW has an antibacterial effect on microorganisms associated with chronic periodontitis	(Nayak et al., 2021)
HW	*S. mutans* and *S. sobrinus*	Glucosyltransferases (gtfB, gtfc, and gtfI) and glucanbinding proteins (gbpC, dblB) (−)	HW can inhibit bacterial growth and biofilm formation	(Liu et al., 2023)
HW	*S. mutans*	gtfB, gtfC (−); *S. mutans* counts and plaque maturation (−)	HW exerts antibiofilm effects on *S. mutans*	(Youn et al., 2025)

## 4 Effects of hydrogen on dental caries

Dental caries, which is the most common oral disease, is caused by multiple factors, mainly bacteria, that induce progressive destruction of dental hard tissues. If not treated in time, it can lead to complications such as pulpitis, periapical periodontitis, and even jawbone inflammation. The existing research on the effects of hydrogen on dental caries is mainly limited to the inhibitory effects of HW on the microbial flora of the dental surface (as described above in Section 3). Common cariogenic bacteria include *Streptococcus mutans*, *Lactobacillus,* and *Actinomycetes.* Kim et al. (2017) found that HW exposure for 60 s remarkably downregulated mRNA expression of glucosyltransferase and glucan-binding protein in *S. mutans* and *S. sobrinus*. Moreover, HW significantly reduced the formation of *in vitro* streptococcal biofilms.

## 5 Effects of hydrogen on periodontitis

**FIGURE 2:**
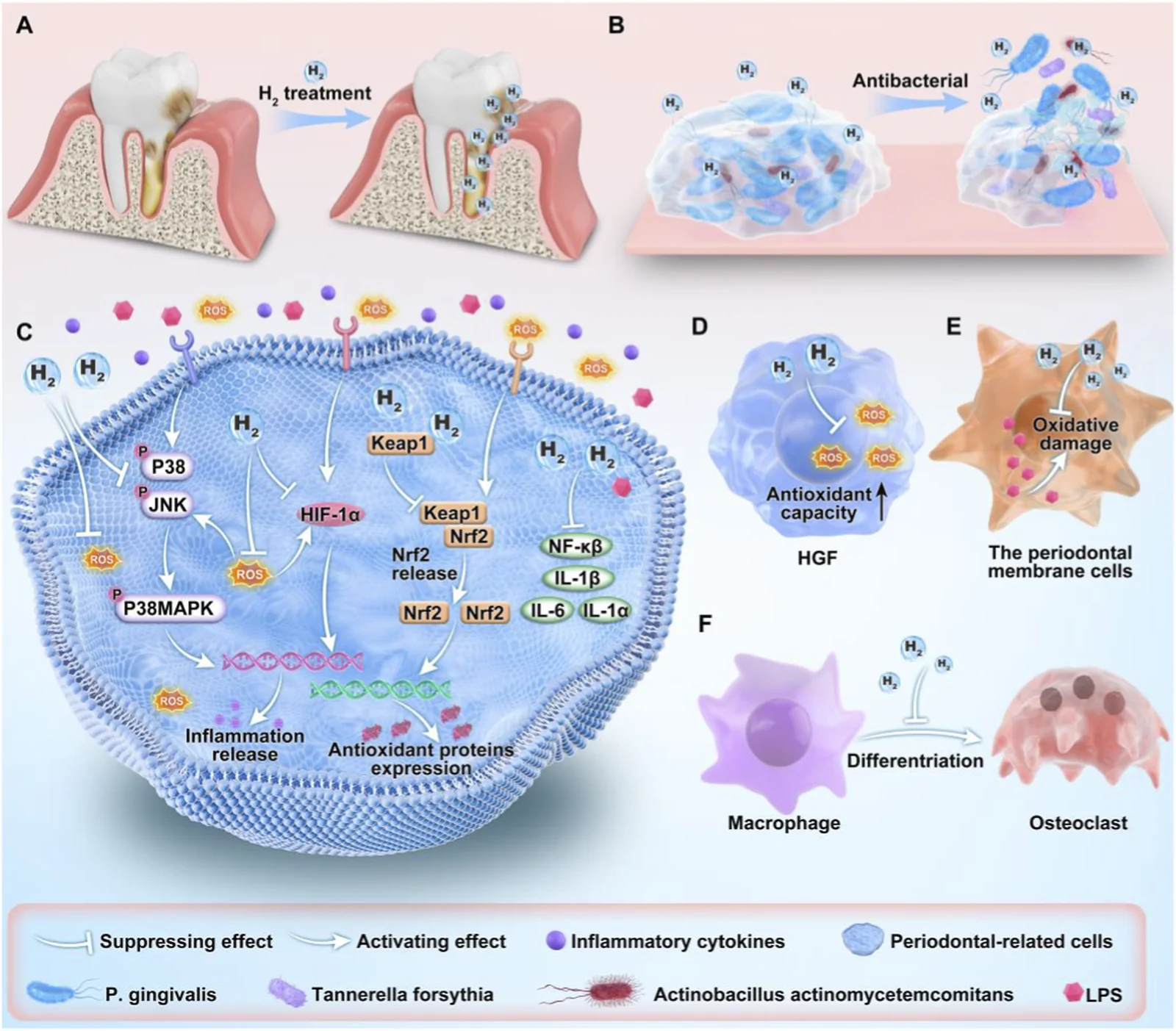
Schematic illustration of the therapeutic effects of hydrogen on periodontitis. **(A)** Hydrogen exerts preventive and therapeutic effects in periodontal tissue. **(B)** Hydrogen inhibits the formation of periodontal pathogenic bacterial biofilms and inhibits bacterial proliferation. **(C)** The signaling pathways through which hydrogen exerts anti-inflammatory and antioxidant stress effects: Hydrogen can suppress the mitogen-activated protein kinase (MAPK) pathway and the ROS/HIF-1α signaling pathway, activate the Keap1/Nrf2 signaling pathway, and inhibit the expression of pro-inflammatory cytokines such as IL-6, IL-1β, IL-1α, and NF-κB. **(D)** Hydrogen can enhance intracellular antioxidant capacity, reduce ROS generation, and protect cells from hydrogen peroxide-induced cell death; **(E)** Hydrogen can protect the periodontal membrane cells and reduce the oxidative stress damage caused by LPS. **(F)** Hydrogen can inhibit osteoclast differentiation following periodontal inflammation.

Periodontitis is a chronic inflammatory and destructive disease of the periodontal supporting tissues that is initiated by plaque microorganisms. It is mainly characterized by periodontal inflammation and alveolar bone resorption, which are major causes of tooth loss across age-groups (de Molon et al., 2026; Hashim et al., 2026). Hydrogen has shown potential preventive and therapeutic effects on periodontitis. While hydrogen is known to inhibit the formation of periodontal pathogen biofilms and bacterial proliferation through its antibacterial actions, it can also exert anti-inflammatory and antioxidant stress effects to inhibit the destruction of oral connective tissue and bone tissue (Bai et al., 2024; Bai et al., 2022). Figure 2 summarizes the mechanisms by which hydrogen may treat periodontitis.

### 5.1 Oxidative stress plays a key role in the development of periodontitis

Periodontal tissue damage is mainly caused by the destruction resulting from the host immune response involving leukocytes, complement factors, and ROS (Yang et al., 2023). The homeostatic imbalance between ROS and the cellular antioxidant defenses triggers an oxidative stress response, causing damage to DNA, proteins, and lipids in host tissues (Yang and Yang, 2025). Periodontitis is associated with a reduction in the total antioxidant status and/or an increase in lipid peroxidation in gingival crevicular fluid, saliva, or blood, indicating that oxidative stress is involved in the progression of periodontitis (Yang and Yang, 2025; Neurath and Kesting, 2024). In the primary stages of periodontal disease, neutrophils are the most common inflammatory cells clustered on tooth surfaces and within the gingival sulcus or periodontal pocket, and they are considered to be the main source of ROS in periodontitis. Furthermore, a substantial body of clinical evidence has suggested that neutrophils mediate an important part of periodontal tissue destruction (Bassani et al., 2023; Qiu et al., 2025). After the stimulation of pathogens, neutrophils produce O_2_^−^ which can be released into the phagosomes and extracellular environment and then converted into different radical and non-radical derivatives, such as hydrogen peroxide (H_2_O_2_) and hypochlorous acid (HOCl) (Yang and Yang, 2025; Shang et al., 2023). Numerous studies have shown that in comparison with the ROS produced by neutrophils in healthy individuals, peripheral blood neutrophils in patients with periodontitis produce more ROS with higher production activity (Uriarte and Hajishengallis, 2022; Mousa et al., 2024). In addition, *in vitro* studies have suggested that under the stimulation of pathogens and/or their components, neutrophils as well as other cells such as monocytes, gingival fibroblasts, and periodontal ligament cells, can enhance ROS production (Mousa et al., 2024; Boşca et al., 2024). The continuous accumulation of ROS further promotes the release of relevant pro-inflammatory factors, such as, IL-1β and IL-6, thereby promoting the sustained development of inflammation (Papadakis et al., 2025).

### 5.2 Hydrogen exerts protective effects against periodontal tissue injury by inhibiting oxidative stress

Hydrogen has been proven to be an effective therapeutic antioxidant that can effectively protect cells and tissues from oxidative and inflammatory damage. Hydrogen could quickly diffuse across membranes, and selectively neutralized hydroxyl radicals and nitrite anions causing oxidative damage without affecting the free radicals involved in cell signal transduction (Chuang et al., 2026). A comprehensive analysis of research data from 25 groups showed that HW effectively increased glutathione peroxidase (GPx) levels and reduced 8-hydroxyguanosine (8-OHdG) levels (Bai et al., 2022). Potential protective effects of hydrogen against periodontitis-related damage mainly manifest as inhibition of gingival and periodontal ligament inflammation as well as osteoclast differentiation. Li et al. (Xiao and Miwa, 2016) investigated the cellular protective effects of HW on human gingival fibroblasts, and indicated that HW could enhance intracellular antioxidant capacity, reduce ROS generation, and protect cells from hydrogen peroxide induced cell death. Hydrogen has been reported to protect the periodontal membrane cells and reduce the oxidative stress damage caused by lipopolysaccharide (LPS) (Liu et al., 2025). Oxidative damage is involved in the differentiation of osteoclasts (Jiang et al., 2025). Kasuyama et al. (2011) showed that drinking HW can inhibit osteoclast differentiation following periodontal inflammation. Tomofuji et al. (2014) demonstrated that drinking HW could effectively suppress the alveolar bone loss caused by aging and/or periodontal inflammation. In addition, hydrogen can inhibit the release of pro-inflammatory factors (Yiwei Zhao et al., 2023).

### 5.3 Hydrogen can regulate cell signaling pathways

The inhibitory effects of hydrogen on periodontitis may be achieved by regulating different cell signaling pathways. For example, hydrogen can inhibit the MAPK signaling pathway, upregulate the expression of genes encoding the Nod-like receptor protein NLRP3 inflammasome, and downregulate the gene expression of nuclear factor (NF)-κB in periodontal tissue (Tomofuji et al., 2014; Kasuyama et al., 2011).

The MAPK pathway is an important signal transduction system in the cell that is involved in cell proliferation, differentiation, apoptosis, and inflammatory responses (Farkhondeh et al., 2020). ROS can activate MAPK pathways, which play an essential role in the development of periodontitis (Yang et al., 2014). Guo et al. (2023) found that hydrogen can decrease the ROS content and reduce the mRNA expression and the levels of phosphorylated p38 MAPK, as well as the level of p38 MAPK. Moreover, hydrogen reduces the number of tartrate-resistant acid phosphatase (TRAP)-positive osteoclasts and significantly decreases the activity of MAP kinases in periodontal tissue, including JNK, p-38, and extracellular signal-regulated protein kinase (Kasuyama et al., 2011).

In the hypoxic microenvironment of periodontium, an increase in ROS levels leads to the accumulation of hypoxia inducible factor (HIF)-1α and the release of various cytokines, thereby activating of the HIF pathway (Fadl and Leask, 2026). Zhao et al. (2020) found that ROS accumulation can activate HIF-1α, thereby driving the pro-inflammatory response of periodontal ligament fibroblasts (PDLFs). Therefore, HIF-1α is a key signaling molecule that controls and alleviates the occurrence and development of periodontitis.

Hydrogen can inhibit the HIF-1α pathway. Hydrogen can suppress the activation of the HIF-1α pathway by reducing ROS. It can also downregulate the expression of HIF-1α to regulate the ROS/HIF-1α signaling pathway, thereby combating oxidative stress and mitigating inflammatory damage. Wei Y. et al. (2023) found that hydrogen may inhibit inflammation by suppressing the release of HIF-1α and IL-1α.

The Kelch-like ECH-associated protein 1/nuclear factor erythroid 2-related factor 2 (Keap1/Nrf2) pathway mediates the principal protective response to oxidative stress and inflammation (Jiang et al., 2024). Molecular hydrogen inhibits the binding of Keap1 to Nrf2 by binding to Keap1 itself, thereby facilitating the release of more Nrf2 to promote this process. The accumulated Nrf2 in the pathway binds to downstream antioxidant response elements and initiates the antioxidant stress response (Hirano et al., 2023).

### 5.4 Hydrogen can inhibit the expression of pro-inflammatory cytokines

Saitoh et al. (2022) investigated the effects of hydrogen on the secretion of several inflammatory markers in human periodontitis models and found that H_2_ could inhibit the production of IL-1α and IL-6. Tomofuji et al. (2014) investigated the effect of HW on the aging of periodontal tissue in healthy rats and found that HW consumption can downregulate the gene expression of NF-κB in periodontal tissue and upregulate the expression of the NLRP3 inflammasome coding genes (i.e. NLRP3, ASC, caspase-1, and IL-1β). These results indicate that drinking HW has anti-aging effects on periodontal oxidative damage in healthy rats.

## 6 Effects of hydrogen on peri-implant inflammation

Peri-implantitis is a major cause of implant failure and cannot be ignored in implant-supported prosthesis. In comparison with periodontitis, the inflammatory cell infiltration area around an implant site is larger and shows more pronounced bone resorption, with the higher proportion of neutrophils and osteoclasts (Huang et al., 2021). Carcuac and Berglundh (2014) found that the mRNA expression levels of IL-6, IL-8, and TNF in the surrounding tissues of implants were higher than those in periodontitis.

Molecular hydrogen can decrease the inflammatory response of the peri-implant tissues and promote tissue integration. In beagle-dog experimental peri-implantitis, local irrigation with HW reduced inflammatory cell infiltration, downregulated pro-inflammatory cytokines (IL-1β, IL-6, MMP-8) and relative abundance of major pathogenic bacterial genera in the peri-implant crevicular fluid (Yiwei Zhao et al., 2023). Yuan et al. (2026) developed a long-lasting hydrogen-releasing implant and found hydrogen can coordinately activate MAPK signaling in both gingival fibroblasts and vascular endothelial cells, modulate macrophage polarization and suppress excessive oxidative stress, thereby promoting peri-implant tissue remodeling.

## 7 Effects of hydrogen on oral mucosal diseases

Oral mucosal diseases (OMDs), also known as soft-tissue oral diseases, are a series of disorders or conditions affecting the oral mucosa and soft tissue. OMDs mainly include oral infectious diseases such as oral candidiasis; oral mucosal patches striae diseases, such as oral lichen planus (OLP); ulcerative lesions or traumatic injuries of oral mucosa, such as recurrent aphthous ulcer; and oral premalignancy, such as oral leukoplakia (OLK) (Gazal et al., 2026; ugli, 2025). The etiology of OMDs is multifactorial and incompletely understood. Few existing studies have evaluated the effects of hydrogen on OMDs, with most studies focusing on the beneficial effects of hydrogen on the healing of ulcerative or traumatic mucosal lesions. Only one case report has described the treatment of OLP using a local HW rinse, indicating that hydrogen may serve as an adjuvant therapy for this type of disease (Shutao and Xiaoqun, 2022).

### 7.1 Oral wounds invovle oxidative stress and the inflammatory response

Acute and chronic trauma caused by surgery, diseases, radiation, chemotherapeutic agents, and injuries are common factors causing damage to the facial skin and oral mucosa. Common oral mucosal injuries include recurrent aphthous stomatitis, traumatic oral mucosal ulcers, and oral mucositis (OM) caused by chemotherapy drugs and radiation. Oxidative stress and the inflammatory response play crucial roles in oral wound pathophysiology. Oxidative stress is a key factor in inflammatory diseases such as recurrent aphthous stomatitis, which leads to wounds in the oral mucosa without a clear underlying cause (Song et al., 2026). Radiation and/or chemotherapy-induced OM (RIOM/CIOM) is an inflammatory or ulcerative lesion of the oral mucosa that is caused by ionizing radiation and/or chemotherapy during tumor therapy. It is the most common and serious complication of chemotherapy and radiotherapy for head and neck tumors (Zhang et al., 2018; Dipalma et al., 2024). Large-scale, deep, and fused ulcers are the most typical clinical manifestations of RIOM/CIOM. The oxidative damage caused by radiation and chemotherapeutic agents can trigger lipid peroxidation, protein oxidation, and the production of double-strand breaks in DNA (Gentile et al., 2021).

### 7.2 Hydrogen enhances the wound-healing process

**FIGURE 3:**
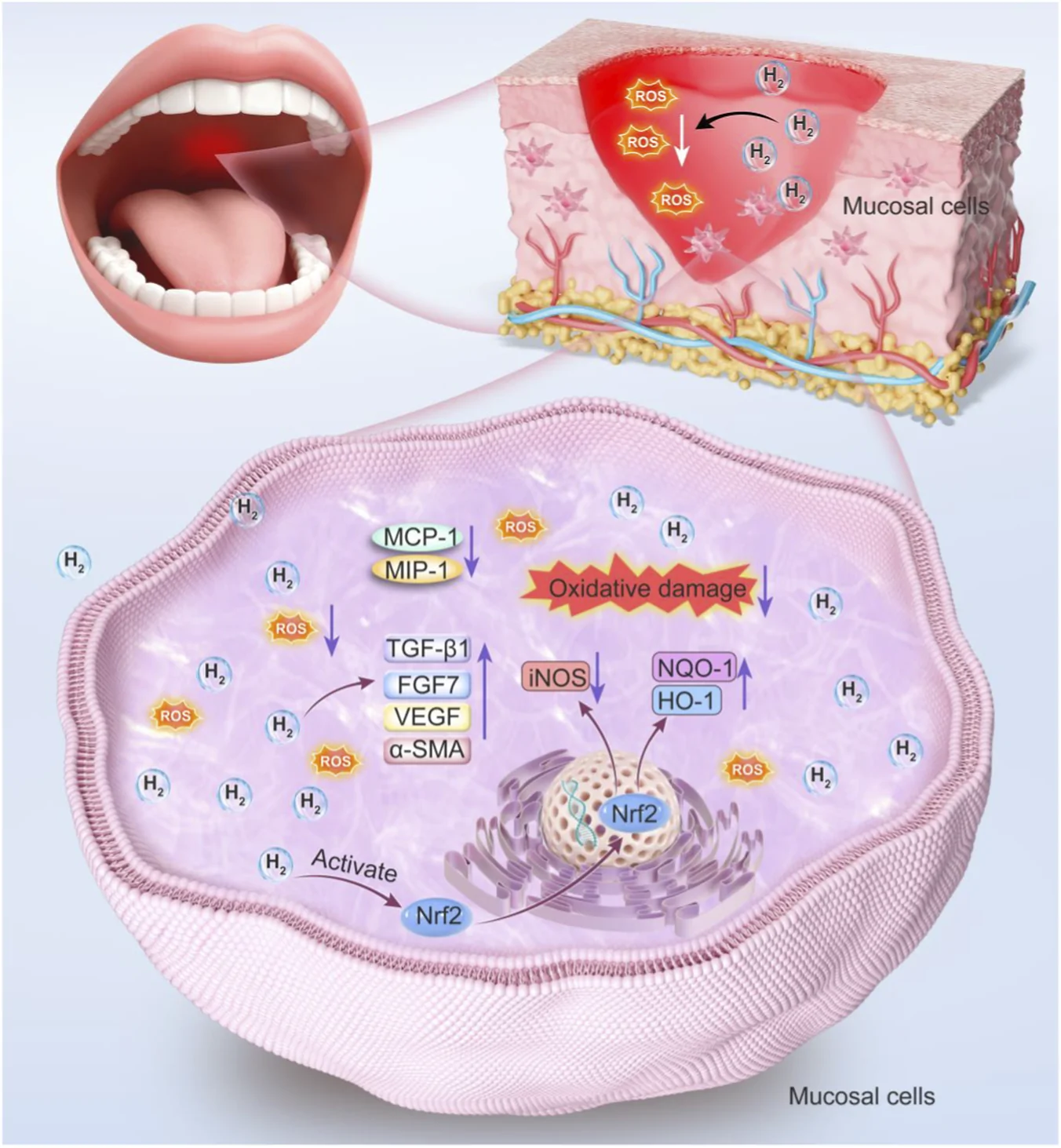
Schematic illustration of the beneficial effects of hydrogen on oral mucosal healing. Hydrogen shows protective effects against oxidative damage. Hydrogen can reduce the levels of pro-inflammatory cytokines (MCP-1 and MIP-1). Hydrogen can activate the Nrf2/antioxidant defense pathway, resulting in increased expression of antioxidant defense genes such as HO-1 and NQO-1. Hydrogen can promote the expression of healing-related genes (TGF-*β*1, FGF7, VEGF, and *α*-SMA).

Hydrogen can promote oral mucosal healing through the following mechanisms (Figure 3): 1) Hydrogen shows a protective effect against oxidative damage caused by radiation, chemotherapy drugs, and trauma. 2) Hydrogen can reduce the levels of pro-inflammatory cytokines, chemokines, and lipid peroxidation. 3) Hydrogen can activate anti-inflammatory defense pathways. 4) Hydrogen can promote the expression of healing-related genes. Tamaki et al. (2016) found that hydrogen suppressed the expression of the pro-inflammatory cytokines monocyte chemoattractant protein (MCP)-1 and macrophage inflammatory protein (MIP)-1 and enhanced the expression of healing-associated genes, such as transforming growth factor (TGF)-*β*1, fibroblast growth factor 7 (FGF7), vascular endothelial growth factor (VEGF), and *α*-smooth muscle actin (SMA). Moreover, hydrogen can activate the Nrf2/antioxidant defense pathway, causing increased expression of antioxidant defense genes such as heme oxygenase (HO)-1 and NAD(P)H quinone dehydrogenase 1 (NQO-1), thereby alleviating systemic oxidative stress and inflammation. Hydrogen also plays an important role in reducing toxicity and enhancing the efficacy of various tumor radiotherapy and chemotherapy regimens (Cui et al., 2024; Lan et al., 2025). Hydrogen has been reported to significantly reduce the severity of radiodermatitis and accelerate impaired wound repair (Kuo et al., 2024).

## 8 Effects of hydrogen on other oral and maxillofacial diseases

### 8.1 Jaw disease

Only a few studies have evaluated the effects of hydrogen on the jaw, and these were limited to the use of HW or hydrogen-rich saline for osteoradionecrosis of the jaw (ORNJ) and osteoarthritis (OA). ORNJ is a serious complication after radiation therapy for head and neck cancers (Corrao et al., 2023). It is usually asymptomatic initially, and is followed by intractable pain, halitosis, dysphonia, and exposed isolation. In the late stage, patients with ORNJ often experience oral mucosal or skin fistulas, pathological fractures, and even life-threatening complications (De Felice et al., 2020; Seo and Kim, 2020). This form of tissue damage caused by radiation exposure has been attributed to inflammation and the formation of large amounts of free radicals, leading to the breakdown of normal tissue structures and causing fibrosis and necrosis (Chrcanovic et al., 2010; Zhao et al., 2007). However, no unified and effective treatment and prevention method for this chronic pathological damage is available at present (Chronopoulos et al., 2018). ORNJ is caused by hydroxyl radicals; therefore, hydrogen, which exhibits strong antioxidant properties, shows the potential to prevent and treat ORNJ. Chen et al. (2020) studied the protective effects of hydrogen on a rat model of ORNJ, and suggested that hydrogen can activate the Nrf2 antioxidant signal, enhance osteoblast differentiation, and promote bone repair and regeneration of mandible after radiation. In addition, to verify the protective effects of hydrogen *in vivo*, they established an ORNJ animal model and found that hydrogen significantly reduced the severity of bone injury.

### 8.2 Oral and maxillofacial tumors

ROS can enhance the survival, proliferation, and migration of tumor cells, contributing to tumor development. Hydrogen can clear intracellular ROS, thereby inhibiting ROS-dependent signaling pathways such as the NF-κB pathway, the heme oxygenase-1 (HO-1) pathway, and other pro-survival pathways that promote proliferation, invasion, and metastasis of tumor cell (Cheung and Vousden, 2022; Meng et al., 2021). In addition, hydrogen can limit the factors contributing to oxidative stress in tumor cells by reducing mitochondrial ROS production (Zhou et al., 2024). An older study by Dole et al. demonstrated that high concentrations of hydrogen could cure squamous cell carcinoma on mouse skin (Dole and Fife, 1975). Hydrogen-rich electrolyzed water has been reported to inhibit ROS levels and proliferation in human tongue cancer and fibrosarcoma cells (Rochette et al., 2021).

### 8.3 Trigeminal neuralgia

Trigeminal neuralgia (TN) is a common facial nerve disease characterized by paroxysmal onset of pain attacks in the facial and mandibular regions where the trigeminal nerve and its branches are distributed (Committee and Headache Classification Committee of the International Headache Society IHS The International Classification of Headache Disorders, 2018; Maarbjerg et al., 2014; Haviv et al., 2016). Demyelination along the trigeminal nerve pathway is the main driving factor for the pathogenesis and pathophysiology of TN (Di Stefano et al., 2019; Chen et al., 2016). In TN, cytokine accumulation and the “ROS storm” generated by the inflammatory cascade can lead to demyelination of the trigeminal sensory roots, tissue swelling, and intense electric-shock-like pain (Zhang et al., 2022; Gao et al., 2025). Hydrogen can alleviate neuropathic pain symptoms in the following ways: 1) Hydrogen can directly clear ROS and inhibit the production of pro-inflammatory cytokines (Kobayashi et al., 2020). 2) Hydrogen can block the NLRP3-caspase-1- gasdermin D (GSDMD) pathway and cytoplasmic pyroptosis, thereby reducing the risk of nerve damage and alleviating pain (Mu et al., 2023; Shi et al., 2015). 3) Hydrogen can regulate the activity and expression of transient receptor potential channels by reducing oxidative stress, altering ion channels and receptors related to pain conduction, and thereby reducing the excitability of nociceptive neurons (Goyal et al., 2023; Li et al., 2023).

## 9 Conclusion

Hydrogen has antioxidant, anti-inflammatory, and cell signaling-regulatory effects and shows great potential in the treatment of oral diseases. Unfortunately, the existing studies have shown the following limitations: 1) the existing research is limited to the cellular level, with few animal and clinical trials; 2) the sample sizes of most of the included studies are small and the follow-up periods are short, thus it is impossible to confirm long-term efficacy and recurrence control effects. Additionally, some differences were observed in the measurement methods and sample sources of various study indicators, which may have led to heterogeneity in the study results; 3) existing hydrogen delivery methods have inherent limitations with no unified standard for effective concentrations, and current approaches struggle to ensure effective therapeutic concentrations and adequate duration of action at oral lesion sites. Therefore, the administration mode and dosage of hydrogen require further study; 4) most of the studies were focused on describing phenomena, and the specific pathways and regulatory mechanisms of hydrogen within the complex oral microenvironment (e.g., microbiota and saliva) have not been fully elucidated, limiting the translation of basic research into precise clinical strategies. Follow-up studies should collect more data and perform subgroup analyses of sample sources, microbial types, and cell types. In addition, in-depth exploration of antimicrobial and anti-inflammatory mechanisms should be conducted to provide a more detailed summary and basis for clinical application.
